# Design of a Real-Time Corrosion Detection and Quantification Protocol for Automobiles

**DOI:** 10.3390/ma15093211

**Published:** 2022-04-29

**Authors:** Kunj Dhonde, Mitra Mirhassani, Edwin Tam, Susan Sawyer-Beaulieu

**Affiliations:** 1 Department of Electrical and Computer Engineering, University of Windsor, Windsor, ON N9B 3P4, Canada; mitramir@uwindsor.ca; 2 Department of Civil and Environmental Engineering, University of Windsor, Windsor, ON N9B 3P4, Canada; edwintam@uwindsor.ca (E.T.); susansb@uwindsor.ca (S.S.-B.)

**Keywords:** corrosion detection, automobiles, light reflectance value, quantification

## Abstract

Corrosion can compromise the integrity of the vehicle. Instead, “rust proofing” a vehicle can prolong its usable life span, reducing material waste overall and permitting greater salvageability at the end of the vehicle’s life. For rust proofing, a definitive and consistent approach for detecting corrosion could be beneficial. Instead, most vehicle corrosion detection and assessment is performed visually and in an ad hoc manner without following any particular guidelines. The visual examination of corrosion depends highly on the method of analyzing and interpreting the corrosion, as well as operator’s experience in assessing and applying rust proofing. As a result, any visual assessment strategy needs standardization to minimize human error. An automated method is proposed to identify and analyze surface rust and appraise its severity for vehicles. The method demonstrated is 96% effective, low-cost, and has low computational complexity. Subsequently, the method has the potential to be conveyed to different advanced devices, such as smartphones, to measure corrosion, decreasing errors and improving measurement accuracy. Low implementation cost, and high reliability of the method contributes to its ease of use in the field, and hence, advances its accessibility to automotive professionals to identify and monitor corrosion levels, without the interference of human errors.

## 1. Introduction

Automobiles operate in a hostile environment that includes gravel, snow, humid weather, and de-icing salts. Although there have been substantial changes and significantly greater awareness of issues related to vehicle performance, safety, and emissions, the impacts of corrosion are still hazardous and can pose short-term and long-term issues. On a consumer level, vehicle corrosion is largely assessed and treated in an ad hoc manner (e.g., when a vehicle owner visits a repair garage). Corroded vehicles can lose their resale value, reliability, safety, and their recoverable materials. This combination poses safety and environmental risks. In 2014, 907 million passenger vehicles and 329 million commercial vehicles were registered globally, compared to 678 million passenger vehicles and 248 million commercial vehicles in 2006. This represents a 33.7 percent increase in passenger car numbers and a 32.6 percent increase in commercial vehicle numbers. If the observed trajectory persists, there will be roughly 1.7 billion registered vehicles on the road worldwide by 2035 [1]. As a consequence, it is important to consider the upkeep of vehicles. Because of the high humidity and use of de-icing chemicals in the northern United States and southern Canada, corrosion is a major concern for automobiles. According to a study prepared for the Federal Highway Administration in 2001, the cost of corrosion in transportation is 29.7 billion CAD, with motor vehicle corrosion responsible for 23.4 billion CAD [2]. Similarly, the US Department of Defense Corrosion Prevention and Control (CPAC) developed a Corrosion Category Code for US army vehicles [3]. However, there are currently a plethora of aftermarket corrosion prevention and treatment products and applications available. At the consumer/aftermarket level, there is no single method for evaluating corrosion that is used consistently through the automobile sector.

Typically, the user of the vehicle or a technician at an automobile facility detects corrosion first through visual inspection, the easiest and most common method of detecting corrosion. Visual inspection, however, is susceptible to human errors. Thus, it is advisable to employ machines for the task if feasible. Different image processing tools were studied that might be useful in anticipating corrosion [4,5,6]. Methods such as edge detection, image segmentation, color analysis, numerical analysis, etc. are the most powerful and low-cost methods to detect corrosion [7,8]. Texture and thickness examination were conducted to accurately identify corrosion [9,10]. All these methods were fast and accurate enough to detect corrosion of one particular type.

While thickness and texture analysis can be used to measure corrosion, a digital imaging-based process represents a realistic balance between the ease of detection and the detail that can be achieved in such exams [11,12]. However, the effectiveness of these methods relies on the noise levels in the image. The effects of corrosion were examined on huge steel infrastructures using computer vision and digital image correlation, respectively [7,13]. Retinex theory was used to recognize corrosion on protective coatings of steel structures [8]. A statistical approach was proposed, which collaborates texture analysis and color analysis to detect the corrosion on steel structures [14].

Texture analysis is a popular, highly attribute-oriented way to detect corrosion and also a way for corrosion monitoring for non-destructive structures [15]. Despite this, there has been not a single texture analysis method that is proven to detect all different textures and colors of corrosion. As a result, texture analysis is not suited to detect vehicular corrosion from a broad perspective. Along with texture and color analysis approaches, deep learning is also employed to analyze corrosion. Wavelet domain, convolution neural networks, and python-based methods have been employed to detect corrosion on huge structures in production plants and infrastructures [16,17].

While several corrosion analysis methods exist, few (if any) have been adopted for consumer and vehicle applications, and many are not designed for practical, in-field use by technicians and operators. The authors propose an automated corrosion detection and quantification method to analyze corrosion and promote future in-field use. The method provides fast and accurate results. In the following section, the procedure used to detect and quantify vehicular corrosion is explained. A dataset is created by sampling 369 vehicles. The dataset is utilized to test and verify the method in Section 3. The conclusion is presented in Section 4.

## 2. Methodology

Vehicle parts differ in their resistance to oxidation. Underbody parts of the vehicle are more prone to corrosion and can, therefore, be exceptionally valuable in detecting the corrosion [18]. As a result, the underbody parts were given primary consideration. The dataset is composed of images captured by inspecting 369 vehicles. The dataset utilizes characteristic images with non-uniform brightening, diverse degrees of deformities, and change in image substance. In this study, noise in the image is referred to all the pixels contributing to non-rusty vehicle parts, such as the wheel and fiber-made parts, garage facility equipment, such as the hoist, and objects present at the facility. In assessing the corrosion on each vehicle, each component of the dataset was categorized into sub-divisions, such as treated or untreated and vehicular age and model. The images were shot using two cameras, Nikon Coolpix P7000 and Canon PowerShot G5X, from a distance of 1.0 m to 1.5 m. A custom-made, yellow-colored “T-scale” ruler was used to capture the images to manually estimate the size of the vehicle part.

**Figure 1:**
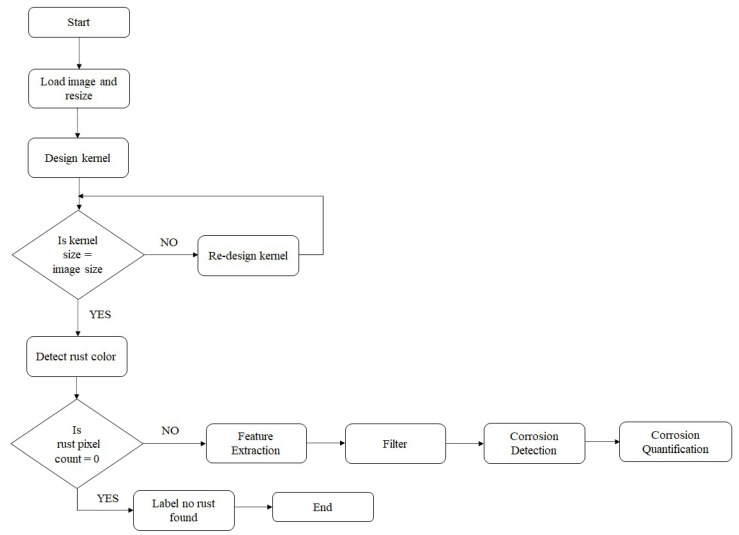
Corrosion detection and quantification algorithm.

It is critical to consistently assess and estimate the images (refer to Figure 1). This ensures that comparison of the corroded region with the non-corroded region was done reliably and without bias. In addition, the images were taken with diverse optical sensors which vary in image dimensionality and resolution specifications. Consideration of different camera resolutions was done in order to make the proposed method robust and independent from the camera specifications. Furthermore, the images used in the research were collected by non-experts, thus causing compromisation of the image quality. The effect of compromised image quality has been discussed in Section 2.2. Considering that every camera lens produces a unique image dimensionality, the images were resized before the corroded region could be recognized. Care was taken to ensure that image resizing did not influence the pixel spatial orientation to avoid the loss of valuable data. Corrosion location was performed by dissecting the rust color and light reflected from the corroded portion. A kernel is designed to check the image and test out the corroded region. Each time the kernel filters a portion of the image, a search for any rust color is performed. In case the color is not found, the image is labeled as a non-corroded image. In case the rust color is found, the image is advanced to examine the corroded zone precisely and further quantified.

### 2.1. Rust Pixel Analysis

A pixel describing the corroded region contains a hue between yellow-orange and dark brown. From the perspective of a visual review, early corrosion can be recognized by the color of the rust, without any requirement for strong investigation. In this manner, the rust color is recognized to identify the corrosion regions roughly.

**Figure 2:**
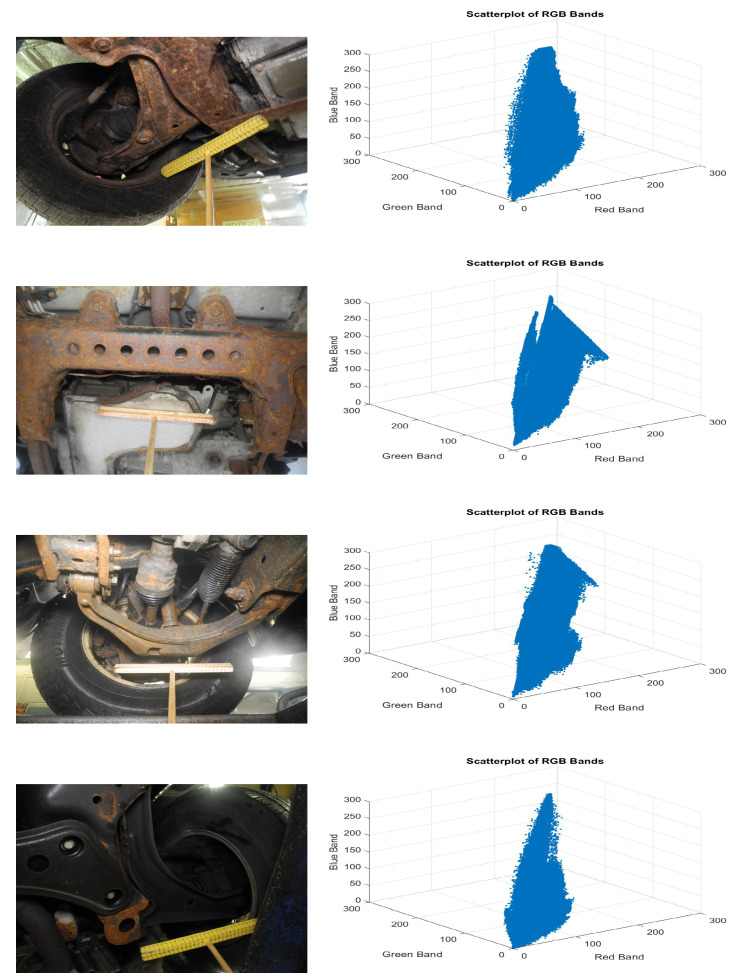
Input images of vehicle underbody parts under different illumination (**left**) and respective correlation visualization among the RGB bands (**right**).

Because the dataset deals with characteristic images, there are numerous colors that are present within the image. These are found to be correlated with the rust color within the RGB bands (refer to Figure 2). To facilitate the exact rust color discovery, all the related RGB bands are de-correlated. This step diminishes the likelihood of wrong rust color discovery. Decorrelation stretch is utilized to extricate the related relationship exhibited among the bands.

In Figure 2, the linear behavior of the scatterplot portrays the direct correlated connection among the RGB bands. The decorrelation stretch may be a process to improve the color contrasts found in a color image by a strategy that incorporates the evacuation of the inter-channel relationship found within the input pixels. Decorrelation improves the color separation of an image with the band–band correlation. The colors of the image helps improve visual interpretation and makes feature extraction simpler. The original color values of the image are mapped to a new color value distribution with an extended range. The color value of each pixel is then transformed into the color eigenspace of the correlation matrix, extending to equalize the band variances. Later, it is transformed to the original color bands.

Decorrelation-stretched images provide an illustration that upgrades unearthly reflectance varieties. The decorrelation stretch is best suited when the input information of all three bands has a joint distribution and an approximate distribution [19]. The adequacy of the decorrelation stretch exceedingly depends on the distribution of the input pixels. In the event that the input distribution is multispectral, the impact of decorrelation stretch may lessen. Luckily, the images utilized for the study are captured utilizing optical sensors working within the visible range.

**Figure 3:**
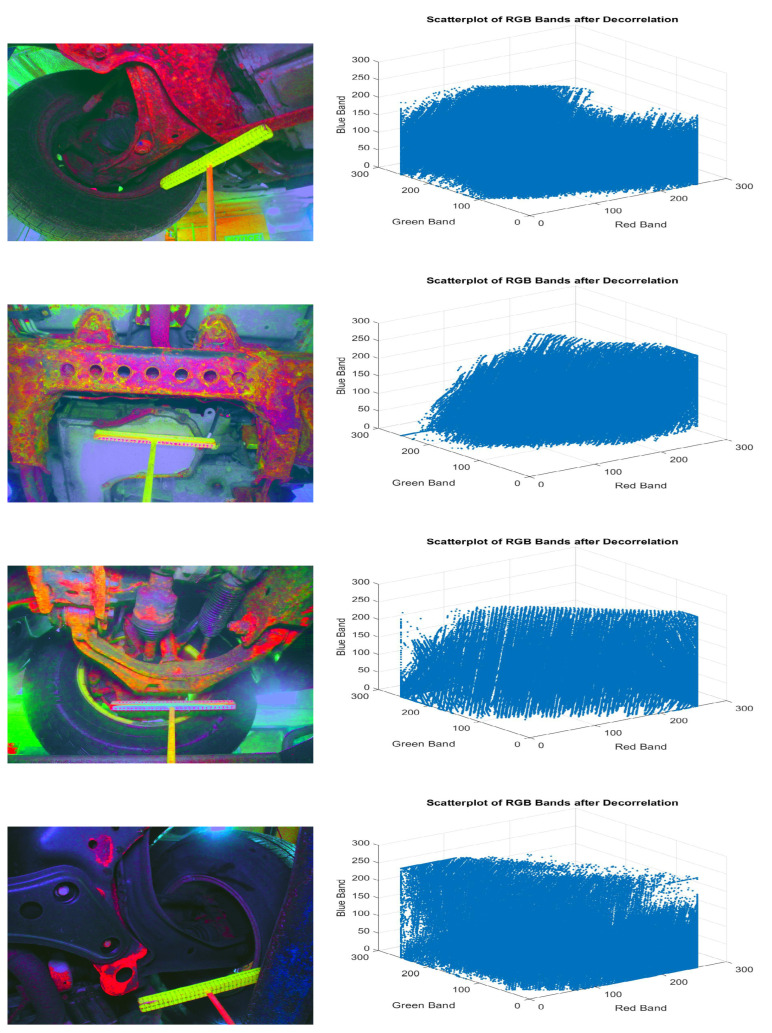
Illustration of Rust Pixel Detection in the de-correlated bands of the original images (**left**) and respective visualization of de-correlated RGB bands (**right**).

Additionally, this step exploits all the inter-band differences that may exist in the image. If the noise content is significant, this step will produce a colorful output with enhanced noisy pixels. In this manner, magnification of the corrosion content in the image is required before finally concluding the precise corrosion detection. The scatterplot within Figure 3 illustrates the decorrelation result. Input distribution within the scatterplot has been consistently spread within the accessible space after the decorrelation step.

Furthermore, the similarity between the images is exceedingly unpredictable. Hence, to precisely detect corrosion pixels despite of the noise, the development of a binary mask utilizing the hue component is proved viable.

Therefore, rust pixels are extracted, masked and highlighted from the rest of the background. A binary mask is produced utilizing the hue component to yield viable masking outcomes. It is reasonable for processing images containing dark or low-contrast zones with undistinguished neighborhood details. However, masking generally extricates the rust pixels and is not dependable completely due to the need of homogeneity within the images.

Figure 3 portrays the input image and the rust pixels location result. It is observed that the rust color has been recognized. Within the rust color detection step, it may so happen that the pixels not contributing to the corroded zone may be recognized as rust pixels. This may happen due to the nearness of noise pixels and presence of undesirable image substance. To arrange to have proficient corrosion detection, it is important to distinguish between corroded pixels and non-corroded ones. The following section explains how the difference is formulated between the corroded and non-corroded zones.

### 2.2. Corrosion Detection

As mentioned, within the RGB space, the unmistakable rust pixels are extricated as the result of the Rust Pixel Detection step. At this stage, it may so happen that dirt or other chemical traces on the vehicle can be recognized as rust. Furthermore, presence of grease/oil along with the limitations of the shooting environment may lead to false pixel detection. This untrue detection leads to wrong corrosion measurement readings. In order to avoid this circumstance, more important data around the rust pixels should be obtained. Hence, the RGB plane has been transformed to HSI (Hue, Saturation, Intensity) plane. Within the HSI plane, data such as brightening level of each pixel and the color each pixel contributes to are portrayed. The intensity component of HSI space describes the different illumination levels in the image. Furthermore, the illumination levels depend on the light reflected by the uppermost surface of the corrosion. This is defined by the term Light Reflectance Value (LRV).

**Figure 4:**
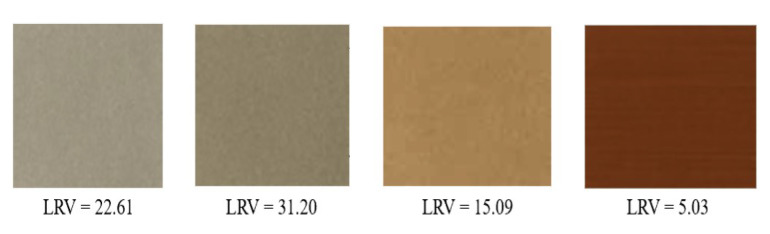
Light Reflectance Values for different surface rust colors.

The surface reflectance of the non-corroded and the corroded portion changes with increasing levels of light within the visible range. On average, the corroded portion is illustrated in the LRV range, with values ranging from 5 to 32 (refer to Figure 4). The LRV values for the non-corroded area ranges from 46 to as high as 70. It is worth mentioning that the LRV value can be influenced by the shooting environment. The image quality and pixel characteristics can be affected if the lighting is not favorable.

**Figure 5:**
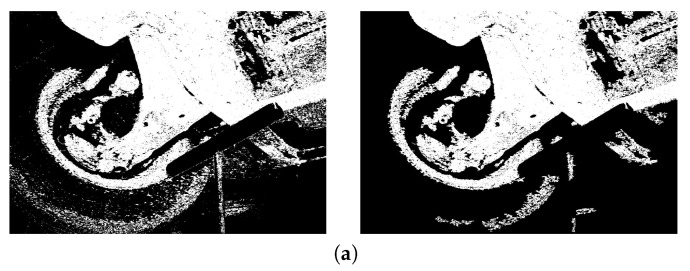

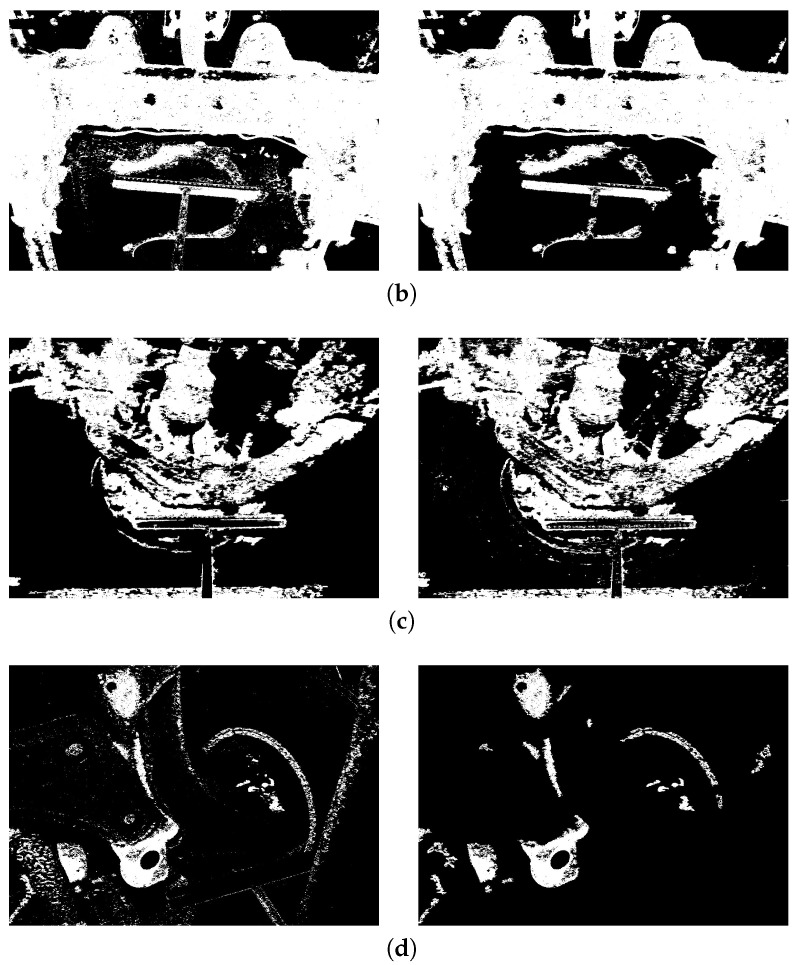
Extraction and quantification of the corroded area after thresholding (**left**) and filtering (**right**). (**a**) Surface Rust Percent = 22.78% ; (**b**) Surface Rust Percent = 67.95% ; (**c**) Surface Rust Percent = 40.17% ; (**d**) Surface Rust Percent = 7.26% .

Based on the rule of light reflectance and illumination levels depicted in HSI space, a threshold is characterized with the most extreme illuminated level for a specific image, which segments the illuminated rust pixels from the rest (refer to Figure 5). Thresholding comes about in upgrade of the contrast levels up to a degree and dispenses with the undesirable color pixels encompassing the rust pixels. Deformity within the yield is further cleaned by employing a median filter (refer to Figure 5).

### 2.3. Corrosion Metrics

Corrosion measurements gives numerical investigation about the corroded region identified. To begin with, rust pixel tally, camera resolution determination, area of the corroded area, and underbody area were utilized to calculate the percentage of the corroded zone [18]. Highlight extraction of identified rust pixels from the image was utilized for corrosion assessment. For each image, rust pixels were examined and checked. The yield is communicated in percentages.

## 3. Experimental Results

### 3.1. Corrosion Detection and Quantification

**Figure 6:**
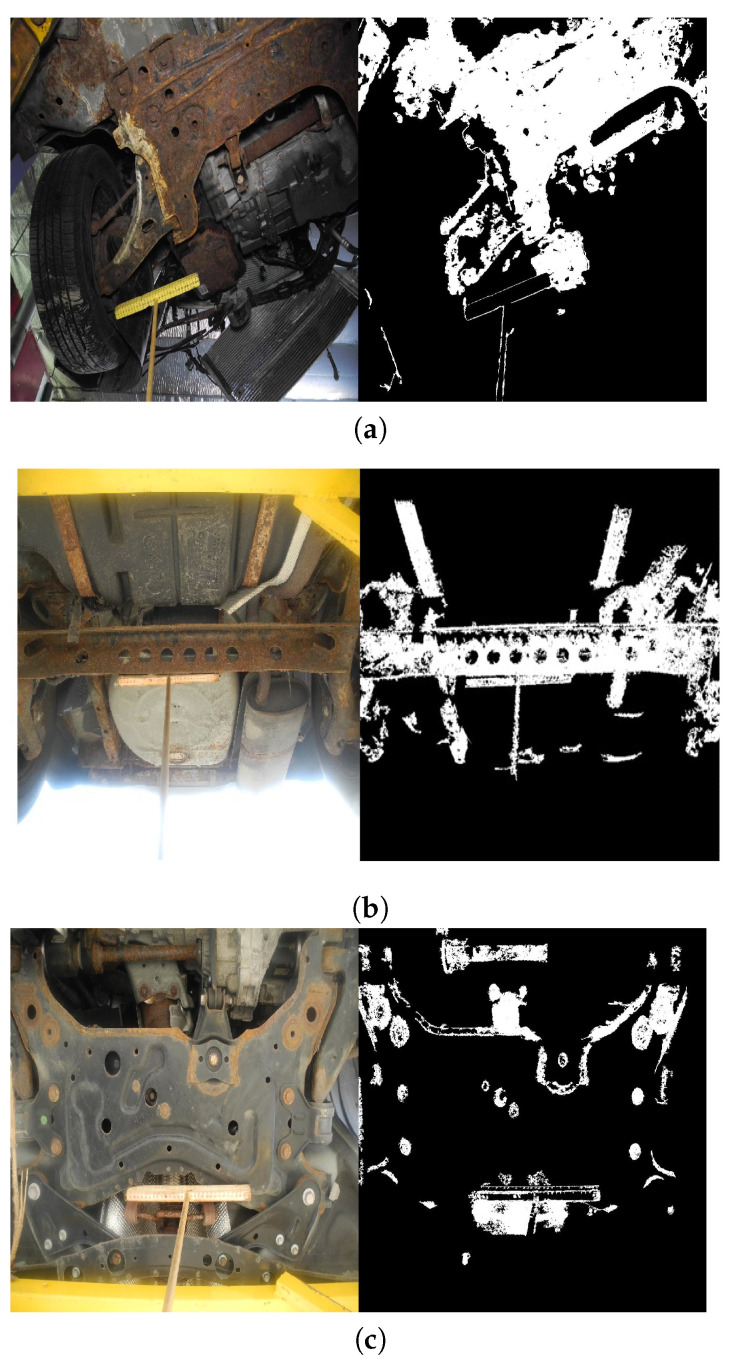
Illustration of corrosion detection and quantification. (**a**) Surface Rust Percent = 39.08% ; (**b**) Surface Rust Percent = 21.42% ; (**c**) Surface Rust Percent = 13.80% .

Corrosion discovery at any stage is crucial to preventing more harm to the vehicle. The proposed calculation is demonstrated valuable to detect vehicle corrosion at any stages (refer to Figure 6). Steel, iron, and aluminum, which make up most of the vehicle body, are all susceptible to moisture in the atmosphere. Oxidation of these metals can be hazardous to the vehicle and the user. Thus, all the above-mentioned materials’ vulnerability to corrosion has been considered in the development of the proposed strategy. Figure 6 demonstrated the proposed corrosion detection method applied to different images taken from the vehicle underbody.

Corrosion can cause the metal to deteriorate, regardless of the metal. Often, corrosion causes the metal to corrupt and gradually breaks into brittle flakes, eventually taking off the metal’s attributes. The flakes’ formation and color change can be taken note of on the metal as soon as the metal begins to oxidize and further advances as the degradation increments. Over time, the oxidized part increases and becomes solid. The proposed method is effective in detecting the cracks, flakes, and solid surface rust formed on the vehicle body due to the corrosion. Moreover, the strategy is also productive to identify common types of vehicle corrosion: surface rust, blisters, and perforation. However, the dimensionality of cracks, crevices, or holes that appear due to stress corrosion was out of the research scope. Additionally, corrosion thickness (depth) calculations were not included in the study.

### 3.2. Performance Evaluation

To evaluate the performance of the proposed method, the relationship between the original image and the output image is determined. As the method’s primary portion is based on the color investigation and images have been compressed before processing, it is prudent to discover the correlation between the original image and processed corrosion detection output image. The attributes of the image has to be preserved at any given point of time. Ten images were selected randomly: five images captured with the Nikon Coolpix and five images captured with the Canon PowerShot camera. For every image, the Pearson Correlation Coefficient was calculated between its original version and analyzed version (refer to Equation (1)). The Pearson Correlation Coefficient is widely used in statistical analysis, pattern recognition, and image processing [20]. The closer two entities are to each other, the higher the correlation coefficient value. Eventually, a table was formulated that demonstrates the behavior of the correlation coefficient among the mentioned entities.

(1) $$\begin{array}{c} Correlation\;Coefficient\;=\frac{\sum_{i}\left(A_{i}-A_{j}\right)\left(B_{i}-B_{j}\right)}{\sqrt{\left(\sum_{i}{\left(A_{i}-A_{j}\right)}^{2}\right)}\times \sqrt{\left(\sum_{i}{\left(B_{i}-B_{j}\right)}^{2}\right)}} \end{array}$$

where

*A* = Original Image;

*B* = Processed Image;

$A_{i}$ = Intensity of *i*th pixel in image A;

$B_{i}$ = Intensity of *i*th pixel in image B;

$A_{j}$ = Mean intensity of image A;

$B_{j}$ = Mean intensity of image B. 

Table 1 represents the degree of linear correlation between the original image and the processed output. The determined correlation coefficient is observed to vary between 0.46 and 1.0, demonstrating a direct correlation.

A distinctive approach was followed to examine the precision of the calculation; for the testing, manual masking of all undesirable image substance was performed. In brief, all the pixels contributing to the things which were not part of the vehicle body were physically blacked out.

**Figure 7:**
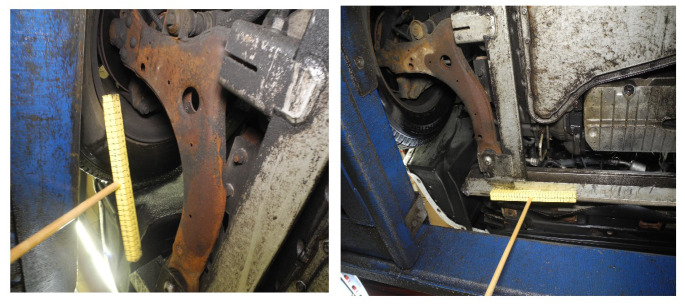
Sample images from dataset 1 consisting of original (unmasked) images.

**Figure 8:**
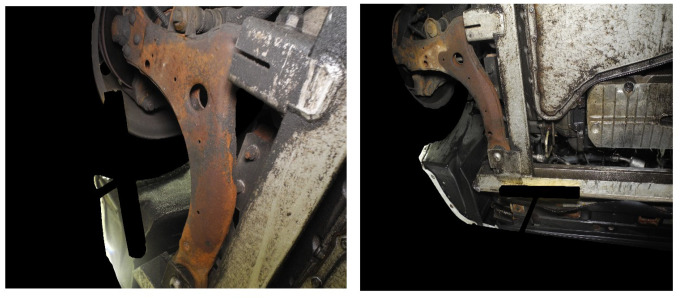
Sample images from dataset 2 consisting of masked images.

Two datasets were considered for the testing: one with crude images (labeled as unmasked images; refer to Figure 7) and the other with masked images (refer to Figure 8). Each dataset has sub-categories, such as treated or untreated and vehicle age groups. Based on the previous studies, vehicle age groups were categorized into four divisions; 0–4 years, 5–8 years, 9–12 years and 13+ years.

**Figure 9:**
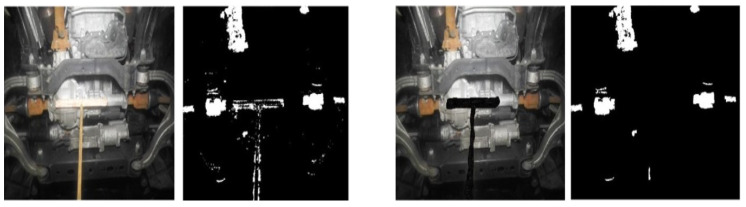
Illustration of corrosion detection in sample images from dataset 1 (**left**) and dataset 2 (**right**).

Figure 9 illustrates the visual difference in the corrosion detection outputs. It can be observed that the removal of undesirable image substance is essential to facilitate accurate algorithm-based corrosion quantification. This action was performed on all 369 images and readings were plotted. All the background objects, such as the wheel (rubber part), rusty garage parts, and garage surroundings, were manually masked.

**Figure 10:**
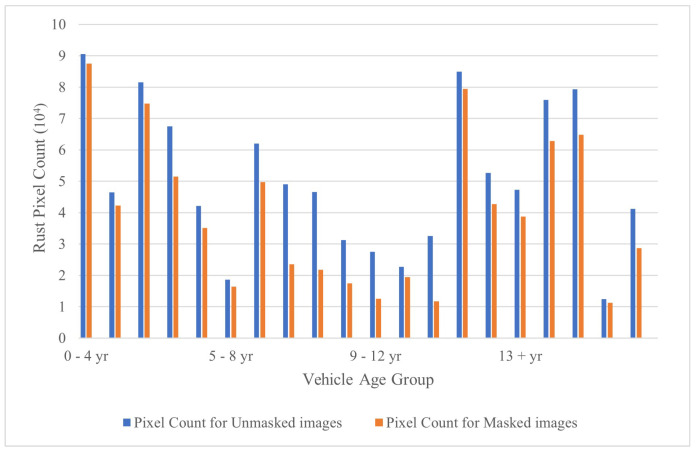
Rust pixel count variations for different vehicle age groups.

**Figure 11:**
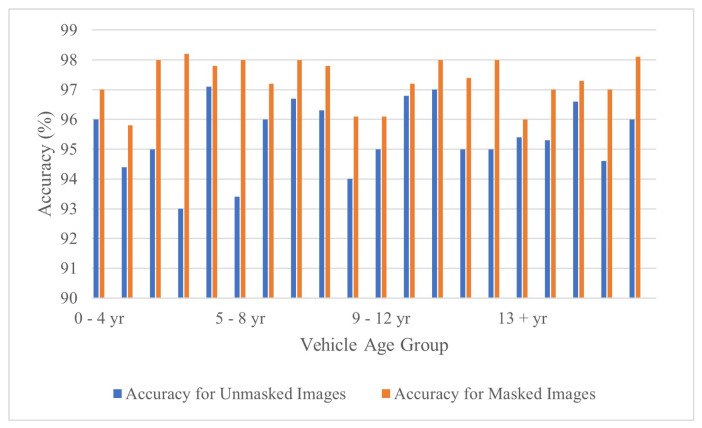
Accuracy tradeoff between the masked and unmasked image dataset.

The rust pixel count was plotted against the vehicle age to study the trend between masked and unmasked rust pixel count. It is worth noting that the distinction number (difference in rust pixel count between unmasked and its masked version) is significant. The rust pixel count is a little higher in the unmasked images (blue color bar in Figure 10) when compared to their masked versions. This implies the method may detect pixels that relate to rust hue. However, the change does not offer a significantly bad impact on the efficiency of the method (refer to Figure 11). The study shows that the method works well with a moderate level of non-uniformities present in the image (refer to Figure 10). Additionally, noise in the image indicates to all the pixels that does not contribute to the vehicular metal body.

This study shows that the method is demonstrated to be close to 98% efficient with moderate noise level images and 93–96% efficient with extremely high noise level images. The difference in efficiencies among the masked and unmasked dataset is altogether considerable. In the study, it was shown that corrosion detection accuracy can be improved simply by focusing on the corroded area in the images.

## 4. Conclusions

The paper centers on detection of vehicular corrosion. Dataset raw images contain an array of issues, such as illuminance heterogeneity, color resolution variance, unfocused areas, dirty vehicle body, and parts of the surroundings (for example, a garage hoist) in which the images were captured. Perceptions and results show that the method works successfully, if not perfectly, in detecting the corrosion for the metals, such as iron, aluminum, and steel. The study shows that the proposed method possesses the potential to detect the common vehicle corrosion types, such as perforations, blisters, cracks, and surface rust. Among the 369 vehicles examined, the results show that the calculation is 96% accurate, with small-scale fluctuation in noise levels. It ought to be said that the calculation cannot realize effective outcomes when the light variations are extremely high and image quality is highly jeopardized. This can be due to the reality that the LRV is exceptionally near to 100%, making it difficult for the optical sensor to distinguish between color shades. In this case, the most elevated achievable accuracy was found to be near to 93%. Performance evaluation shows that the method is more efficient when the image content is more focused on the corroded area. The authors are working to optimize the efficiency by facilitating the automated masking of the undesired pixels (all pixels that do not describe vehicle parts) in the image. This will make in-field technicians’/users’ lives easier and eliminate the issues of poor image quality and lack of focus. We conclude that the algorithm has the potential to be a viable corrosion detection tool for use in the automotive industry.

## Figures and Tables

**Table 1:** Demonstration of correlation between original images and their respective corrosion detection output images.

Dataset Image Name	Vehicle Age (in Years)	Camera Used	Treated/Untreated	Correlation Coefficient
IMG_0852.JPG	6	Canon	Treated	0.85
IMG_8370.JPG	18	Canon	Untreated	0.58
IMG_9313.JPG	19	Canon	Untreated	0.79
DSCN1568.JPG	5	Nikon	Treated	0.46
DSCN3857.JPG	12	Nikon	Treated	0.97
DSCN3731.JPG	13	Nikon	Untreated	0.92
DSCN1745.JPG	17	Nikon	Treated	0.99
IMG_0687.JPG	23	Canon	Treated	0.83
IMG_8055.JPG	16	Canon	Treated	0.79
DSCN3460.JPG	16	Nikon	Untreated	0.99

## Data Availability

The data presented in this study are available on request from the corresponding author. The data are not publicly available due to the proprietary nature of the vehicle images and the integrally-related vehicle owner’s information and survey data.
